# The efference cascade, consciousness, and its self: naturalizing the first person pivot of action control

**DOI:** 10.3389/fpsyg.2013.00501

**Published:** 2013-08-09

**Authors:** Bjorn Merker

**Affiliations:** Kristianstad, Sweden

**Keywords:** action control, attention, consciousness, egocentric space, first person, pulvinar, self, superior colliculus

## Abstract

The 20 billion neurons of the neocortex have a mere hundred thousand motor neurons by which to express cortical contents in overt behavior. Implemented through a staggered cortical “efference cascade” originating in the descending axons of layer five pyramidal cells throughout the neocortical expanse, this steep convergence accomplishes final integration for action of cortical information through a system of interconnected subcortical way stations. Coherent and effective action control requires the inclusion of a continually updated joint “global best estimate” of current sensory, motivational, and motor circumstances in this process. I have previously proposed that this running best estimate is extracted from cortical probabilistic preliminaries by a subcortical neural “reality model” implementing our conscious sensory phenomenology. As such it must exhibit first person perspectival organization, suggested to derive from formating requirements of the brain's subsystem for gaze control, with the superior colliculus at its base. Gaze movements provide the leading edge of behavior by capturing targets of engagement prior to contact. The rotation-based geometry of directional gaze movements places their implicit origin inside the head, a location recoverable by cortical probabilistic source reconstruction from the rampant primary sensory variance generated by the incessant play of collicularly triggered gaze movements. At the interface between cortex and colliculus lies the dorsal pulvinar. Its unique long-range inhibitory circuitry may precipitate the brain's global best estimate of its momentary circumstances through multiple constraint satisfaction across its afferents from numerous cortical areas and colliculus. As phenomenal content of our sensory awareness, such a global best estimate would exhibit perspectival organization centered on a purely implicit first person origin, inherently incapable of appearing as a phenomenal content of the sensory space it serves.

## Introduction

“Given the presumption that the way we see the world evolved to make the control of action as straightforward as possible, it is likely that our phenomenal perception of the world is closely related to the mechanisms we use to act upon it”

Michael Land (Land, 2012, p. R811).

Whatever a theory of consciousness might contain or propose, it must provide an account of what it is that places us in a *first person* perspectival relation to our phenomenal experience. So central is this relation to the constitution of the conscious state that it virtually defines it (Velmans, 1991; Merker, 1997). This much at least is certain, without such an account a theory cannot be adequate to the greater part of ordinary waking reality, because in it we routinely experience the events of our lives. The “we” here refers, of course, to the “first person” in question. Neither self-consciousness nor a self-image is implied by this usage; to be subject to phenomenal experience suffices. To the extent that any notion of self is consciously entertained, it shares with other items or contents of consciousness the status of being apprehended from a first person perspective. The latter does not, in other words, presuppose self images or self-consciousness, but they presuppose it.

To be explored in what follows is the proposition that the first person perspective, and with it consciousness, is best understood in relation to the requirements of action control (Merker, 2005, 2007; Land, 2012), and has its origin in them. It is there that one finds the key to the kinds of content that enter the conscious state (Morsella, 2005) and also the functional grounds for the peculiar tripartite nested format in which the first person perspective of our sensory consciousness is cast (Merker, 2007, 2013). In this endeavor we shall be concerned almost exclusively with sensory consciousness, and visual sensory consciousness in particular. This is not because other domains of conscious contents are without interest, but because nowhere is the first person perspective more concretely defined, more instructively instantiated, or more empirically accessible than in immediate phenomenal sensory experience.

Sensory experience is typically treated on the afferent side of cerebral operations, concerned with how the brain interprets and makes sense of the barrage of irregular spiking activity arriving on its sensory nerves. Action control, on the other hand, is typically treated on the efferent side, presupposing that the world has been deciphered, and one is ready to act upon it. The *apparent* contradiction of making action control the key to sensory experience stems from conflating sensory operations—the ramified activity of the cortical sensory hierarchies—with sensory experience. The latter is conscious, and the phenomenal objects that populate it bear no trace of the massive multi-stage operations the cortex mounts in order to strip them of the multiple dimensions of inherent ambiguity encumbering the brain's primary afference (see Merker, 2012 and references therein). Sensory objects present themselves to our consciousness as finished products of the cortical hierarchies, delivered on completion of their labors (which accordingly may take place unconsciously).

There are, moreover, good grounds for believing that the cortex employs a probabilistic data format for its many internal operations (Hinton and Sejnowski, 1983; Földiák, 1993; Anderson and Van Essen, 1994; Zemel et al., 1998; van Rossum et al., 2002; Pouget et al., 2003), and that our sensory world is a running *global best estimate* based upon those probabilistic cortical preliminaries (Merker, 2012). The cortex, furthermore, has reason to avoid precipitating final estimates within its own operations (van Rossum et al., 2002; Merker, 2012; see Beck et al., 2008 and Ma et al., 2006 for an example). It is perfectly feasible, then, to entertain the possibility that the implementation of our sensory awareness takes place in structures among efferent targets of cortical operations, provided they have the requisite representational capacity and are in receipt of direct projections from a suitable set of cortical areas. What such an arrangement might look like when pursued into the targets of descending cortical pathways will be explored in the sections that follow.

## The efference cascade defined

It is an all too common misconception that cortical control over behavior is exercised principally through direct projections from primary motor cortex to the motor neuronal apparatus of lower brain stem and spinal cord, and that the rest of the cortex influences behavior indirectly, via its typically multisynaptic transcortical connections to primary motor cortex. But no cortical area is dependent on the motor cortex for its efference^1^ because every cortical area has direct subcortical projections descending from pyramidal cells populating its lower two cortical layers (Diamond, 1979; Jones, 1984; Thomson and Lamy, 2007).

One contingent of these descending projections issues from cortical layer VI to “near” (often reciprocally connected) subcortical structures such as the thalamus and the claustrum [reviewed in Thomson (2010)]. In the thalamus they exert a merely modulatory influence on their target structures via small boutons synapsing on distal dendrites and engage the thalamic reticular nucleus (likewise modulatory) by collaterals when passing through it (Guillery, 1995; Erişir et al., 1997; Sherman and Guillery, 1998; Prieto and Winer, 1999; Rouiller and Welker, 2000; Li et al., 2003; Wang et al., 2006). In the setting of reciprocal corticothalamic connectivity this large population of layer VI cells presumably is engaged in “tuning” neural activity on its way *up to* the cortex (cf. Ferster and Lindström, 1985; Martin and Somogyi, 1985; Li and Ebner, 2007; da Costa and Martin, 2009), whether that activity originates in thalamic sensory relay nuclei or higher order ones.

It is cortical layer V, however, that contains pyramidal cells engaged in exporting cortical information to distant targets, and therefore can be expected to convey a final summary of cortical operations to the rest of the brain. It supplies numerous diverse and far-flung subcortical targets in basal ganglia, basal forebrain, diencephalon, midbrain, pons, medulla, and spinal cord with typically high-security driving synaptic input via large boutons that synapse on proximal dendrites (Kuypers, 1981; Jones, 1984; Guillery, 1995; Sherman and Guillery, 1998; Rouiller and Welker, 2000; McHaffie et al., 2001; Winer, 2006; Lemon, 2008). *Every cortical area issues such descending projections*. Their precise subcortical targets depend on the cortical area in question. In this laminar sense, then, all of cortex can be said to have a motor function (Diamond, 1979; cf. also Jones, 1984, p. 522; Campbell, 1905; Bolton, 1910; Swanson, 2000).

Not all long descending cortical projections terminate in motor related structures, however. Some innervate brainstem sensory structures such as the trigeminal sensory and dorsal column nuclei (Kuypers, 1981). *Here the term “efference cascade” will therefore be used as a comprehensive and functionally neutral term for the entire diverse system of descending (extra-telencephalic) cortical layer V projections*. It originates in large pyramidal cells concentrated to lower cortical layer V.

These layer V pyramids exceed all other cortical cell types in the comprehensiveness with which they sample activity across cortical layers (Larkum, 2013). Their basal dendrites often extend into cortical layer VI below them (e.g., Dégenètais et al., 2002 Figure 10; Ledergerber and Larkum, 2010, Figure 12), and their robust and typically branching apical dendrites extend as prominent tufts into the supragranular layers including layer I. Special conductance and spike initiation mechanisms operate to connect this tuft compartment with the basal dendrite and axon initial segment compartment via action potential backpropagation (Amitai et al., 1993; Yuste et al., 1994; Larkum et al., 1999, 2004; Larkum, 2013). They thus appear ideally disposed to issue a comprehensive summary to the rest of the brain of the state of the local patch of cortex in which they reside.

It was in this sense that Douglas and Martin summarized their role as follows, “The pyramidal cells of layer 5 that drive subcortical structures involved in action (e.g., basal ganglia, colliculus, ventral spinal cord) decide the output of the cortical circuits” (Douglas and Martin, 2004, p. 443). The axons of these pyramidal cells do not send collaterals to the thalamic reticular nucleus even when passing through it on their way to the dorsal thalamus (Jones, 2002). This, in present terms, is in keeping with their operational role as conduits for the running record of *completed cortical labors* rather than earlier operational stages requiring tuning of activity *arriving* at cortex from subcortical sources.

**Figure 1:**
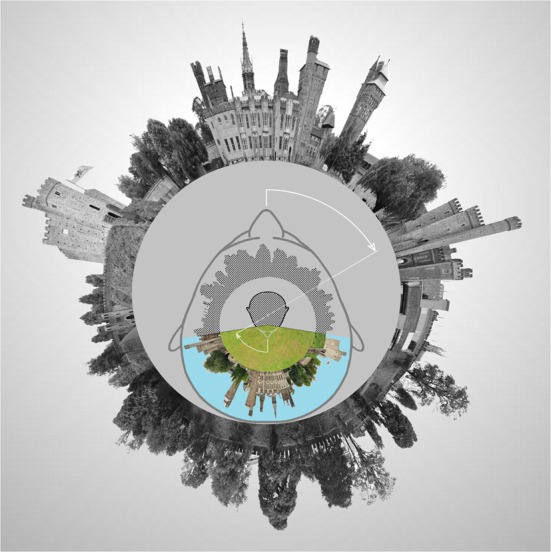
**Polar panorama of Cardiff Castle surrounding an observer head, to illustrate the use of an egocentric neural representation of ambient space in the control of rotational displacements of eyes and head during orienting movements.** Only a head movement is depicted. For inclusion of eye movements in such a scheme, see Land (2012). The physical universe is rendered in gray scale, while the contents of the neural reality model (shown filling the physical head only to gain image resolution) are rendered in color and raster. **Colored sector:** The visible portion of the surroundings representated in the neural reality model, anchored to the perceptual egocenter inside the reality model's head representation (rastered, because not within the field of view). **Rastered sector:** The remaining multimodal space representation of the neural reality model, tacitly present for vision in the form of sectors of ambient space that may be brought within the field of view by gaze displacements. In such a scheme perceived angular distance to a potential orienting target matches the required rotational displacement of the physical eyes and head (gaze), symbolically indicated by the line joining the two angular displacements. The execution of such a movement is *experienced* as a movement of one's (i.e., neural model) *head only*, while one's (i.e., neural model) surroundings *remain stationary*, though the *physical* surroundings undergo wholesale displacement relative to the sensory receptors fixed to the moving physical eyes and head in the course of that movement. The tacit representation of the surroundings (rastered) accordingly must undergo a corresponding compensatory displacement in the neural reality model, leaving the rastered sector “locked,” as it were, to the physical surroundings despite head movements, presumably in dependence on oculomotor efference copy and vesitibular head movement signals (see further, Land, 2012). The content of the colored sector, of course, is always what is before the eyes. For gaze movements from one primary position to another that content always occupies the same fixed sector of the reality model (i.e., without requiring translatory movement), given surround compensatory movement plus saccadic suppression. In the proposed dorsal pulvinar implementation of such a reality model, the compensatory surround movement can draw on afference from both colliculus and posterior parietal cortex, the latter in receipt of disynaptic hippocampal, cerebellar and collicular (Clower et al., 2001), as well as vestibular (Andersen, 1997), information. The Cardiff Castle panorama photo is from Gregg M. Erickson under a Creative Commons Attribution 3.0 Unported license, modified in polar coordinates by Nevit Dilmen under the same license, further modified for inclusion in this figure by Bjorn Merker and released under the same license.

The morphological and physiological specializations of layer V pyramidal cells ensure that the spiking activity of their axons comes to reflect the overlap in time of activity across cortical layers (Jones, 1998; Douglas and Martin, 2004; Larkum, 2013, box 1, Figure 1; Thomson et al., 2002). They appear to be particularly well disposed, in fact, to reflect conjoint activation of cortical feedforward and feedback projections in their activity (Larkum, 2013). This circumstance carries special significance for the present topic, because a number of lines of evidence suggest that such conjoint activation is a condition for cortical information to enter consciousness (Lamme and Spekreijse, 2000; Bullier, 2001; Merker, 2004, p. 566 and Figure 4; Lamme, 2010; Boly et al., 2011).

It is conceivable, therefore, that somewhere beneath the cortex there is a target or set of targets of these cortical layer V pyramidal cell axons in which their “reporting” on the cortical pattern of conjoint activation of feedforward and feedback activity becomes conscious, after passing a threshold in that subcortical terminus. Combined with the reasons alluded to in the previous section for provisionally excluding the cortex itself as a venue for precipitating the sensory estimates that yield phenomenal perceptual objects (a full rationale is presented in Merker, 2012), it seems worth exploring the distinct possibility that the brain's mechanism of consciousness might hide among targets of cortical layer V descending projections.

## Picking a path through the wilderness

The massive many-to-few convergence by which the efference cascade connects vast expanses of cortex to compact subcortical nuclei is an appropriate design feature for a system that derives concise final estimates from cortical probability distributions for purposes of action control. Given that no more than roughly a hundred thousand motor neurons must execute every behavior influenced by some 20 billion cortical neurons, a steep convergence ratio is a systemic necessity. This fits well with the modest representational requirements of final estimates compared to their capacity-intensive probabilistic preliminaries (Ma et al., 2006; Beck et al., 2008). Here, however, we are not concerned with just any estimate, but with the brain's global best estimate of its current circumstances, proposed to fill our consciousness with the world we experience around us (Merker, 2012, 2013). Do compact subcortical nuclei have the neuron numbers and representational capacity to accomodate such content?

A calculation based on a well-studied aspect of phenomenal sensory content, namely visual acuity as a function of eccentricity, discloses that some 164,000 picture elements (“pixels”) suffice to render a monochromatic, monocular, full-field human visual percept at full psychophysical (i.e., phenomenal; see Rock, 1997) resolution (Rojer and Schwartz, 1999; see also Lennie, 1998, p. 900, and Watson, 1987). By rough extrapolation from this measure, a few million neurons employed as representational elements should readily accomodate the full compass of multimodal human sensory awareness (for additional detail, see Merker, 2012, p. 49). This in turn means that a number of the way stations of the efference cascade, such as the superior colliculus in the midbrain and the mediodorsal nucleus as well as the pulvinar complex of the higher order thalamus have the requisite neuron numbers to do so (for cell counts, see Théoret et al., 2001; Abitz et al., 2007; Chalfin et al., 2007).

At least on this score, then, the search for an implementation of a mechanism of sensory consciousness among the subcortical targets of the efference cascade can proceed without embarrassment. In so doing, the *generic structural characteristics of phenomenal sensory consciousness* can be used to canvass the tangled anatomy of the search space for candidate implementing mechanisms (Merker, 2012, 2013). So far this phenomenal resource remains curiously under-exploited in consciousness theory^2^, though it would seem to be a necessary requirement for any matching of candidate neural mechanisms to the operational requirements of the function they are conjectured to implement.

One of the more conspicuous structural characteristics of sensory experience is the nested arrangement in which it comes to us. The world we inhabit is laid out before us in consciousness as a three-dimensional panorama surrounding a central object, our body, from which we look out upon the world through an empty opening in its upper face region (Mach, 1897; Merker, 2007, 2013). The key claim of the present proposal is that this nested egocentric organization of sensory consciousness is inherently related to and derived from the needs of action control in that it simplifies the conversion of locational differences in phenomenal space to directional displacements in our most ubiquitous category of behavioral output, namely the targeting movements of spatial orienting behavior (Hassler and Hess, 1954; Sokolov, 1963; Johansson et al., 2001; Land, 2012). Subsequent sections will expand on this theme, but for now a minimal sketch of the rudiments of an egocentric orienting system is provided in Figure 1.

Gaze or orienting movements account for a greater share of behavioral variance than any other kind of movement. They typically provide the temporally leading edge of all instrumental acts by landing on the targets of those acts *ahead* of the implementing body part (for detail, see Merker, 2012, pp. 46–47). The strategy applies all the way down to the split-second details of manipulative activity (Johansson et al., 2001). Arm and fingers *follow* the agile movements of the gaze as if attached to it by elastic bands. The coupling of arm or hand to the gaze appears to be the brain's default mode of operation (Gorbet and Sergio, 2009; Thaler and Todd, 2009; see also Lünenburger et al., 2001; Reyes-Puerta et al., 2010; Crawford et al., 2011), and so called gain fields (Andersen and Mountcastle, 1983; Chang et al., 2009) can be likened to the “elastic bands” in the analogy just used.

These leading gaze or orienting movements accordingly can be regarded as the brain's principal output. To a first approximation they consist of rotary displacements of the eyes in their orbits and of the head on its cervical pivot. Rotation-based coordinate transformations accordingly are central operations in their coordination and control (Crawford et al., 2011). That control is implemented by highly conserved and complex sensorimotor circuitry of the brainstem (Simpson et al., 1988; Büttner-Ennever et al., 1989; Grantyn et al., 1992; Masino, 1992; Isa and Sasaki, 2002), ultimately anchored to the vestibular system (Cohen, 1988). All higher control of orienting behavior must in one way or another access that control circuitry.

The circumstances just reviewed allow a considerable portion of the efference cascade to be put to one side for present purposes. In his comprehensive survey of the “anatomy of the descending pathways” of 1981, Kuypers identified two major contingents of these pathways (Kuypers, 1981). He called them Group A and Group B. The fiber tracts of Group A follow and contribute to the brain's most basic and earliest formed fiber tract, the medial longitudinal fasciculus (Ross et al., 1992). Through this contingent of medially descending tracts, vestibular, oculomotor/reticular, tectal and other fiber systems effect a set of spatially directional motor adjustments that regulate the body's basic postural orientation to its surroundings in gravitational, inertial, and other spatial sensory system terms (i.e., the functional domain outlined in Roberts, 1973). This medial system is crowned by the control circuitry for eye, head, and (in many species) ear movements that together with trunk movements determine the direction of gaze during orienting movements (Hassler and Hess, 1954; Henkel and Edwards, 1978; Büttner-Ennever et al., 1989; Grantyn et al., 1992; Masino, 1992; Isa and Sasaki, 2002; Horn, 2006).

The fiber tracts of Group B descend in a lateral course through the brainstem, and functionally supplement those of Group A with motor adjustments centered on distal extremities such as those involved in manipulative activity. Group B circuitry accordingly can be thought of as the part of the efference cascade by which the brain guides the body's “engagement” with the configuration of a selected target object or event, while Group A “orients” the body to its global surroundings and targets within it. There is an obvious match between these two contingents of the efference cascade and the “leading” and “following” components of behavior referred to above. It is only the first of these movement domains, those of orienting, that are served by the simplifying geometry of egocentric, rotation-based transformations reflected in the nested format of our sensory consciousness. The search space for a hypothetical implementation of sensory consciousness within the targets of the efference cascade accordingly can be confined to components of Kuypers' Group A “orienting” circuitry.

Even then, Group A features daunting complexity, and further constraints are needed. Functionally, a unitary displacement of the gaze from one target location to another is typically effected by a minimum of two partly independent but linked motor systems, those of eyes and head. The most caudally located premotor site for unitary specification of gaze displacements is the superior colliculus in the roof—tectum—of the midbrain [(Munoz et al., 1991; Freedman et al., 1996; Freedman and Sparks, 1997; Scudder et al., 2002); reviewed in Sparks (2004); see also (Khan et al., 2009)]. Downstream from the superior colliculus the circuitry for control of eyes and head again diverge (Masino, 1992; Scudder et al., 2002; Sparks, 2004; Horn, 2006).

The search for a unitary global best estimate mechanism can be confined, in other words, to targets of cortical layer V projections concerned with orienting behavior located between the cortex and the isthmic caudal border of the midbrain. Within this territory, the numerous targets of descending projections from the principal orienting-related cortical areas, namely the frontal eye fields and gaze-related partietal cortex in primates (Huerta et al., 1986; Stanton et al., 1988a; Saint-Cyr et al., 1990; Lock et al., 2003) are entangled in intricate mutual connective relations within which an ordering principle is nevertheless discernible. As pointed out by Huerta et al. (1986, pp. 434–435), the colliculus belongs among the more prominent targets of both of these cortical areas, and many of their *other* subcortical targets—typically connected with one another—project to the colliculus and are targeted by the colliculus in its turn. The functional significance of this curious parallel or duplicative connectivity will be explored in what follows.

## An orienting superhub in the roof of the midbrain

**Figure 2:**
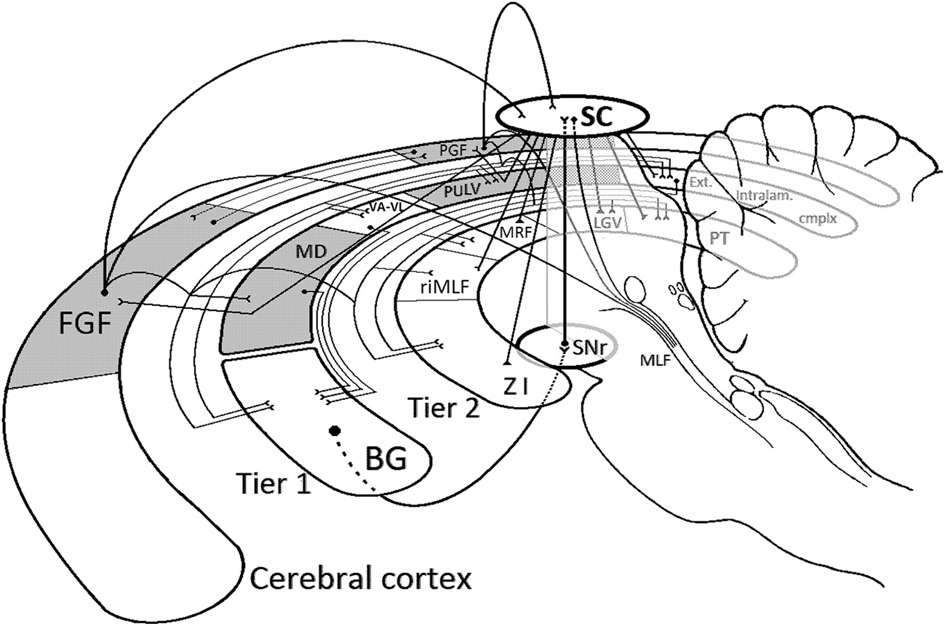
**Schematic depiction of the basic connective relations of the supranuclear apparatus for gaze control discussed in the text.** The figure may conveniently be inspected by proceeding from the two principal cortical “gaze fields,” the frontal (FGF) and the parietal (PGF), which are mutually connected. Projections descending from them are shown as curvilinear trajectories, further distributed to components of Tier 1 [dorsal thalamus and basal ganglia (BG)] and Tier 2 (ventral thalamus and midbrain) via connective “buses” (for graphical economy). Connections between components of Tiers 1 and 2 are omitted to avoid clutter, with two exceptions: Tier 1 projections destined for the basal ganglia (BG) are shown, as are the main connections of both tiers with the superior colliculus (SC). Both cortical gaze fields issue direct projections to the colliculus as well as to the brainstem orienting apparatus. The latter has a token representation in Tier 2 in the form of its most rostral member, the rostral interstitial nucleus of the medial longitudinal fasciculus (riMLF). Connections to the rest of that apparatus are shown descending along the medial longitudinal fasciculus (MLF), and include the direct collicular descending projections to the paramedian brainstem and spinal cord. The colliculus returns projections to the cortical gaze fields via synapses in the paralaminar MD (MD) and Pulvinar (PULV), shown as straight lines deflected in the respective dorsal thalamic nuclei. Note, finally, that the chief descending route from Tier 1 to the brainstem orienting apparatus proceeds from the basal ganglia (which also receive direct cortical gaze field projections) via its midbrain outpost, the substantia nigra pars reticulata (SNr), to the superior colliculus. Together with the rest of its connectivity sketched here, this places the colliculus in the position of connective superhub in the supranuclear apparatus for gaze control, a concept further explicated in the text. Solid dots mark the source of a projection. The termination of a projection is shown ending in an open “Y.” Filled triangles indicate reciprocal connections. Ext. intralam. cmplx, extended intralaminar complex, which includes the suprageniculate and limitans nuclei; VA-VL, ventral anterior and ventrolateral nuclei; PT, pretectal nuclei; LGV, ventral lateral geniculate nucleus (pregeniculate of primates); MRF, midbrain reticular formation; ZI, zona incerta. The figure was inspired by the passage on pp. 435–436 of Huerta et al. (1986). For further detail, see Goldman and Nauta (1976); Fries (1984); Asanuma et al. (1985); Lynch et al. (1985); Leichnetz and Goldberg (1988); Selemon and Goldman-Rakic (1988); Saint-Cyr et al. (1990); Shook et al. (1991); Lock et al. (2003); May (2006); and Stanton et al. (1988a,b).

At least half a dozen areas of the macaque cortex have functional specializations related to the control of gaze movements (see Lynch and Tian, 2006 for a detailed treatment). Of these, the principal ones are the frontal eye fields inside the arcuate sulcus of the frontal lobe and the parietal gaze area in the lateral bank of the intraparietal sulcus, henceforth “cortical gaze fields” for short. The telencephalic, diencephalic, and mesencephalic targets of descending projections from the cortical gaze fields are shown in barest outline in Figure 2, along with some of the principal connections among those targets. Together these structures form the basic supranuclear apparatus for control of gaze (orienting) behavior between the cortex and the mesopontine isthmus. It is interposed, in other words, between the cortex and the brainstem reticular and cervical spinal motor circuitry for eye and head movement control. In the figure they have been grouped into two “subcortical tiers.” One contains cortical gaze field targets in basal ganglia and dorsal thalamus, and the other their targets in ventral thalamus and midbrain.

Tier 1 consists of the gaze field recipient zones in the striatum and a paramedian constellation of orienting-related thalamic nuclei which in addition to the pulvinar complex includes what might be called the “extended intralaminar complex.” The latter is a set of thalamic nuclei that share the property of projecting to the basal ganglia (Powell and Cowan, 1956; Jones, 1989, 1998; McFarland and Haber, 2000, 2001). They include the suprageniculate and limitans nuclei at the caudoventral border of the thalamus, the parafascicular, central lateral, and paracentral nuclei of the classical intralaminar nuclei (weakly connected to the gaze fields) and (flanking the paracentral nucleus) “paralaminar” portions of the mediodorsal, ventral anterior, and ventral lateral nuclei.

The striatal destination of many of the projections issuing from dorsal thalamic targets of the cortical gaze fields, along with the direct gaze field projections to the striatum, makes the basal ganglia the center of gravity of Tier 1 projections. This is reinforced by the fact that the chief thalamic targets of the cortical gaze fields lack descending projections of their own. Thus, as far as orienting gaze behavior is concerned, the principal descending exit from Tier 1 (i.e., from dorsal thalamus and striatum) is through the basal ganglia output pathway for gaze-control. It passes via the substantia nigra pars reticulata and lateralis in the ventral midbrain to the superior colliculus in the roof of the midbrain (Beckstead et al., 1979; Hikosaka and Wurtz, 1983, 1989). As the main connecting link between the first and second tiers, the substantia nigra of the midbrain occupies a position of its own in Figure 2.

Tier 2 has its most rostral outpost in the zona incerta, a ventral thalamic derivative on the undersurface of the dorsal thalamus (see Merker, 2007, pp. 75–76). It further contains the ventral lateral geniculate nucleus (the pregeniculate nucleus of primates; also a ventral thalamic derivative), the anterior and posterior pretectal nuclei, the rostral interstitial nucleus of the medial longitudinal fasciculus, as well as the midbrain reticular formation and what might be called the “perioculomotor nuclei” (the interstitial nucleus of Cajal, nucleus of Darkschewitsch and nucleus of the posterior commissure, not represented in the figure). Finally, it contains as its most elaborate and prominent member the superior colliculus in the roof of the midbrain. Anatomical references are cited in the legend to Figure 2.

The various components of Tier 2—unlike a number of those in Tier 1—have descending projections of their own. In the case of the gaze-related output of the substantia nigra—the principal conduit for the entire descending output of Tier 1—this projection terminates in the intermediate layers of the superior colliculus. This makes the superior colliculus the principal, if indirect, premotor output station of Tier 1. In addition to conveying the output of Tier 1, the colliculus receives prominent direct projections from the cortical gaze fields themselves (Huerta et al., 1986; Stanton et al., 1988b; Lock et al., 2003), as well as from a number of their Tier 2 targets. This convergence of gazefield-related connectivity on the superior colliculus is complemented—as pointed out by Huerta and colleagues and illustrated in Figure 2—by collicular projections to virtually the entire gamut of their diencephalic and midbrain targets (Huerta et al., 1986, pp. 435–436).

Apparently the superior colliculus occupies a central position in the descending connectivity of the cortical gaze fields, suggestive of “superhub” status in informal graph theoretic terms. Assigning it such a role by no means implies that the superior colliculus constitutes an obligatory link in the descending gaze field control over eye and head movements. Instead it opens the possibility that it may perform a more indirect or higher order function than its midbrain location might suggest. It is but one of many subcortical targets of the cortical gaze fields. Among these, most Tier 2 structures have independent descending brainstem projections, and the cortical gaze fields themselves project beyond the midbrain to brainstem nuclei with functions in the control of eye end head movements (Schiller et al., 1980; Schnyder et al., 1985; Huerta et al., 1986; May and Andersen, 1986; Stanton et al., 1988b; Faugier-Grimaud and Ventre, 1989; Shook et al., 1990, 1991; Munoz and Schall, 2004), though some of these projections are not very strong.

In this setting, a collicular role as connective superhub means that from virtually any component of the supranuclear orienting apparatus sketched in Figure 2 there typically is a short synaptic route to the superior colliculus and via it to any other component of that apparatus. *The range of collicular connective relations, arrayed in tandem (i.e., in parallel) with the complex orienting circuitry it serves, seems to indicate that the superior colliculus performs a central function which otherwise diverse components of that circuitry have reasons to access and presumably derive benefit from*. What might that function be?

## The key to collicular function

**Figure 3:**
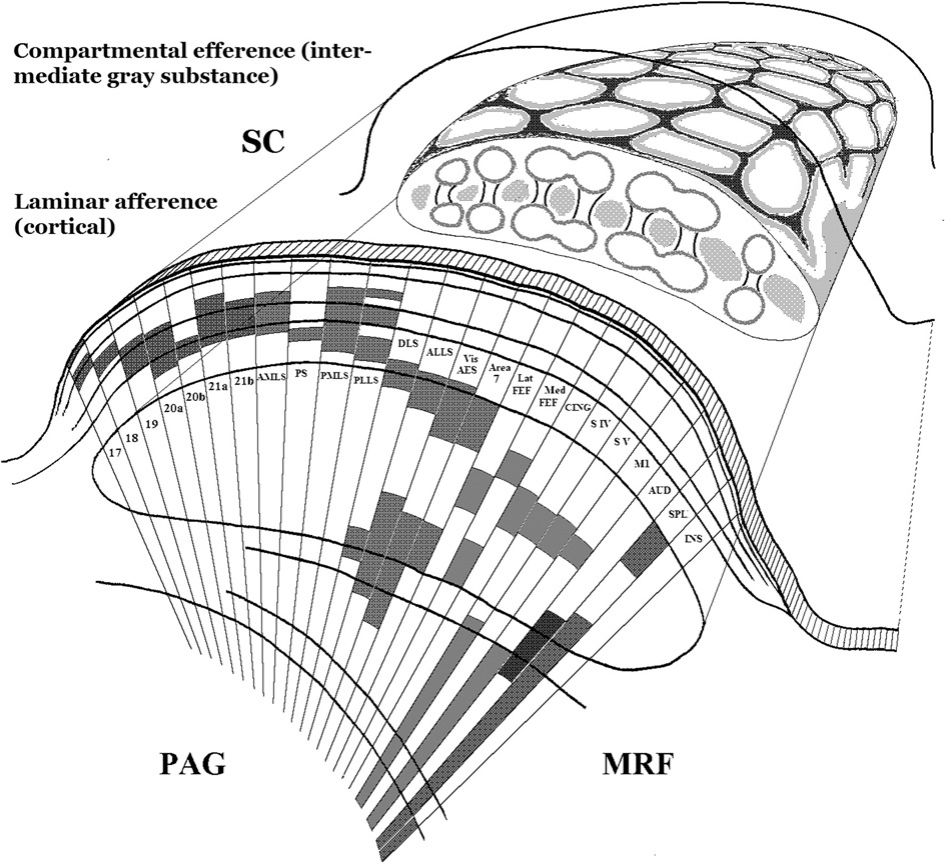
**Schematic depiction of two principal design features of the anatomical organization of the superior colliculus. Lower left:** The cortex-like segregation, by laminar depth in the colliculus, of collicular afferents from many and diverse cortical and subcortical sources. Here only cortical sources are illustrated. Each source typically projects through the full mediolateral extent of the colliculus, but is here shown only as a narrow sector in which its laminar depth is marked by shading. The drawing is a simplified adaptation of results by Harting and colleagues in the cat (Harting et al., 1992), patterned after their summary Figure 27. **Upper right:** A cartoon of the compartmental organization of the collicular intermediate gray substance, based on histochemical and connectional studies in rat and cat (Harting et al., 1992, 1997; Chevalier and Mana, 2000). The upper surface of the composite drawing is patterned after Figure 6 of Chevalier and Mana (2000), and its cut face is loosely patterned after Figure 26 of Harting et al. (1992). Note that this part of the figure combines patterns from rat and cat, and is not anatomically veridical. It is only intended to convey the honeycomb-like tessellation of the collicular intermediate gray substance, by means of which distinct input-output “channels” are concatenated within a shared sensori-motor topography. See further the studies just cited, as well as Deniau et al., 2007 and Redgrave et al., 1992. SC, superior colliculus; PAG, periaqueductal gray matter; MRF, midbrain reticular formation.

The wide-ranging afferent and efferent connectivity of the superior colliculus indicates that it must perform an integrative function of wide scope. A multitude of sensory as well as non-sensory cortical and brainstem systems converge with laminar specificity on its layered structure in the roof of the midbrain (see Figure 3). In the cat more than 40 subcortical nuclei and over 25 cortical areas project to it (Edwards et al., 1979; Edwards, 1980; Harting et al., 1992; see also Grofová et al., 1978; Hikosaka and Wurtz, 1983; Huerta and Harting, 1984; Rieck et al., 1986; Canteras et al., 1994). Collicular output, in turn, distributes divergently: not only do its descending projections target a range of brainstem systems controlling the diverse effectors of orienting movements, including those of the ears in animals that move them (Henkel and Edwards, 1978), but contrasting behavioral output categories are functionally segregated within them (Dean et al., 1988, 1989; Moschovakis et al., 1988a,b; Westby et al., 1990; Redgrave et al., 1993; Mana and Chevalier, 2001; Comoli et al., 2012). Its ascending projections, meanwhile, target the telencephalon (cortex and basal ganglia) via the higher-order and intralaminar thalamic nuclei, as already outlined (Huerta and Harting, 1984; Sparks and Hartwich-Young, 1989; May, 2006).

Well over a century of behavioral and physiological studies indicate that this integrative hub somehow serves the multi-effector phasic movements that re-orient an animal's receptor surfaces relative to a spatial target of immediate behavioral interest (Adamük, 1870; Hassler and Hess, 1954; Schneider, 1967; Schaefer, 1970; Syka and Radil-Weiss, 1971; Straschill and Rieger, 1973; Goodale and Murison, 1975; Harris, 1980; Merker, 1980; Roucoux et al., 1980; McHaffie and Stein, 1982; Milner et al., 1984; Dean et al., 1989; Freedman et al., 1996; Gandhi and Katnani, 2011). The canonical form of this re-orienting is the swift and seamlessly integrated joint action of eyes, ears (in many animals), head, and postural adjustments that make up what its pioneering students called the orienting reflex (Sokolov, 1963)^3^. Collicular involvement in this central pivot of behavior extends even to its autonomic and cerebral activation aspects (Jefferson, 1958; Dean et al., 1991; Dringenberg et al., 2003).

It would be tempting to call the colliculus the “central pattern generator of the orienting reflex,” were it not for the fact that it does not actually specify the particular moment to moment sequence in which eyes, ears, head, trunk or limbs combine to produce a given orienting movement. The interplay among components of orienting gaze shifts is apparently settled downstream of the colliculus (Sparks, 2004). There the elaborate brainstem connectivity bundled along the medial longitudinal fasciculus carries the vestibular, cerebellar, and postural information, including eye position information, integral to the fluid interplay of the several effector organs involved (Büttner-Ennever et al., 1989; for the complexities involved in eye-head coordination alone, see Crawford et al., 1999; Sparks, 1999; Scudder et al., 2002).

Moreover, the behavioral role of the colliculus is not confined to the orienting reflex as classically conceived. Without a colliculus, animals do not exhibit escape reactions to visual threat (Sprague et al., 1961; Denny-Brown, 1962; Sprague and Meikle, 1965; Casagrande and Diamond, 1974; Merker, 1980; Dean et al., 1989; King and Cowey, 1992). Such escape behavior re-orients the animal *away from* the eliciting stimulus, and no orienting *toward* that stimulus need precede the precipitous escape triggered by an effective visual threat (Merker, 1980)^4^. Again, the escape behavior itself is presumably orchestrated downstream of the colliculus, with involvement of the nucleus cuneiformis and periaqueductal gray matter located directly beneath the colliculus (Sprague et al., 1961; Blanchard and Blanchard, 1987; Dean et al., 1988).

Functionally, there is little common ground between orienting target acquisition and escape from visual threat except this: in both situations the brain selects a “spatial target of immediate behavioral priority” toward which the animal's receptor surfaces are re-oriented. In the case of escape behavior, that spatial target is a safe place or escape route and not the eliciting stimulus itself—in fact, the farther from that stimulus the better! A so far elusive generic definition of collicular function may accordingly come within reach by focusing on the *determination of target priority* rather than on either the eliciting stimulus or the nature of the resulting movement (see Schall and Thompson, 1999; Fecteau and Munoz, 2006; Boehnke and Munoz, 2008).

Such a function, it is hereby proposed, may be formulated as follows: *The superior colliculus provides a comprehensive mutual interface for brain systems carrying information relevant to defining the location of high priority targets for immediate re-orienting of receptor surfaces, there to settle their several bids for such a priority location by mutual competition and synergy, resulting in a single momentarily prevailing priority location subject to immediate implementation by deflecting behavioral or attentional orientation to that location*.

The key collicular function, according to this conception, is the selection, on a background of current state and motive variables (Dorris et al., 2007), of a single target location for orienting in the face of concurrent alternative bids. In this capacity the colliculus would serve as the brain's final “priority comparator” or “priority gate” for immediate re-orienting. It would determine which of simultaneous bids for an orienting movement (including that of continuing the current orientation unchanged, Munoz and Guitton, 1989; Peck, 1989) should prevail in gaining momentary control of collicular output circuitry housed in its intermediate layers. The colliculus resolves conflicts, in other words, between the many brain systems whose state bears on an impending orienting movement. According to one theory of the function of phenomenal states (Morsella, 2005), this should give it a role in the constitution of such states. What that role might be is a question the present analysis is laboring to answer.

To clarify further the priority gate function of the collicular orienting superhub: what will be impaired in the absence of the colliculus is not eye or orienting movements as such—as orienting superhub the colliculus is arrayed both in parallel and in series with cortical gaze fields (see Figure 2 and Schiller et al., 1980)—but the process of selection among concurrent bids for target location priority. Depending on task and situational particulars this may take the form of deficient selection and triggering of alerting, orienting and escape reactions—impaired distractibility being a common symptom of collicular lesions across species (Denny-Brown, 1962; Casagrande and Diamond, 1974; Goodale et al., 1975, 1978; Milner et al., 1978; Merker, 1980; Albano et al., 1982; Gaymard et al., 2003)—or impaired ability to regulate orienting priorities in a learning situation (Winterkorn and Meikle, 1981).

Selection of the spatial target for the next orienting movement is not a matter of sensory locations alone, but requires access to situational, motivational, state, and context information determining behavioral priorities. It combines, in other words, bottom-up “salience” with top-down “relevance.” As emphasized by Munoz and colleagues, priority is a weighted combination of these two types of information (Fecteau and Munoz, 2006; Boehnke and Munoz, 2008). This provides a rationale for non-sensory collicular afference such as that originating in cortical association areas and hypothalamus, and more generally the conspicuous convergence of exogenous (bottom-up) and endogenous (top-down) information sources in the superior colliculus (cf. Lines and Milner, 1985; Rieck et al., 1986; Cooper et al., 1998; Trappenberg et al., 2001; Felsen and Mainen, 2008; Reyes-Puerta et al., 2009; Cohen and Castro-Alamancos, 2010; Meeter et al., 2010; Maior et al., 2012).

No cortical gaze field is as directly connected to as wide a range of sources carrying information bearing on the decision where to turn next as is the midbrain superior colliculus. The cortical gaze fields receive high level information but not primary sensory afference, while the colliculus receives both the latter and the direct output of the cortical gaze fields and numerous additional cortical and brainstem afferents as well. Its broader afference enables its intrisic circuitry to weigh a wider range of information bearing on the very next orienting movement than any other known neural system [with the possible exception of the zona incerta, with which it is reciprocally connected (Merker, 2007, pp. 75–76)]. This predicts that without a colliculus an animal will be capable of turning and orienting, but not with as comprehensive a moment-to-moment *weighting and comparison/gating* of all relevant sources of information as when in possession of an intact collicular hub.

The intricate intra- and inter-laminar circuitry within the colliculus that carries out the requisite interactions among its many inputs is beyond the scope of this review [(Moschovakis et al., 1988a,b; Doubell et al., 2003); see review in Isa and Hall (2009)]. Suffice it to say that it involves massive inhibitory interactions, both intrinsic to the colliculus (Katsuta and Isa, 2003) and coming from outside in the form of powerful inhibitory projections from several sources, only one of which is the already mentioned nigral projection. They include the zona incerta, anterior and posterior pretectal nuclei, the periparabigeminal area, a “critical zone” of the pedunculopontine region, and indirectly, via collicular interneurons, the parabigeminal nucleus (Ficalora and Mize, 1989; Appell and Behan, 1990; Behan and Appell, 1992; May et al., 1997; Durmer and Rosenquist, 2001; Klop et al., 2006; Lee and Hall, 2006). Through this convergent interface, multiple functionally diverse systems—each occupying a unique laminar depth in the colliculus—have their say, via inter- and intralaminar collicular interactions, in the moment to moment determination of the next priority target location.

The advantage of conducting structured interactions between low-level primary afference and high-level cortical information in a compact, convergent, laminar mechanism is twofold. First, this way the brain escapes the liability of entrusting moment-to-moment decisions to an executive fed only highly derivative information. When high-level cortical areas place their priority bids with an independent priority comparator, the brain as a whole, through its offices, stays open to “last split-second” course corrections, even by low-level sensory information, provided its magnitude is sufficient to override current competitors (cf. Marino et al., 2012). It is worth noting in this connection that cognitively demanding high-level deliberations are often readily postponable in comparison with intrusive sensory change that might spell disaster unless immediately attended to. Though often a fleeting glance is all that is required before ongoing behavior can be safely resumed, these “cautionary glances” nevertheless compete with the demands of ongoing behavioral task execution. Both utilize the same effector equipment for orienting, hence the need for a mechanism to resolve conflicts between them (Morsella, 2005; see Goodale et al., 1975 for an example).

Second, by taking place in a compact neural space by means of short axon intrinsic connectivity, the interactions needed to determine target location priority can occur far faster than anything that might be accomplished by long-range cortico-cortical interactions among multiple systems. The abolition of short latency gaze shifts by lesions of the colliculus or its local inactivation (Schiller et al., 1980, 1987; Hikosaka and Wurtz, 1985, 1986) accordingly may reflect the absence of the rapid descision making competence by which the colliculus normally drives the orienting machinery (Yarbus, 1967; Sparks et al., 2000; Johansson et al., 2001; Schiller et al., 2004), rather than a mere quantitative slowing of the orienting system.

There is thus no need to interpret the oft reported “vacant stare” and “fixed gaze” of colliculectomized tree shrews and monkeys (Denny-Brown, 1962; Anderson and Symmes, 1969; Casagrande and Diamond, 1974; Keating, 1974; Butter, 1979) as a symptom of an inability to move the eyes or to orient. Rather, without the broad-based afference and rapid operation of the collicular decision making machinery the incessant lively play of the orienting reflex triggered at the collicular interface of endogenous and exogenous signals is compromized, leaving orienting behavior impoverished (see citations on impaired distractibility above).

Among investigators reporting impoverished orienting behavior in monkeys after lesions centered on the superior colliculus, none was more impressed by the lack of spontaneity in post-lesion behavior than was Derek Denny-Brown. In his Sherrington memorial lecture of 1962 he reported on the behavior of five macaques with such lesions, stressing a global deficit in spontaneous behavior as a key symptom of their brain damage. The animals showed a “gross reduction in all types of externally directed behavior,” spent long periods “staring aimlessly into space,” and uttered no sounds (Denny-Brown, 1962, pp. 536–537). These global deficits appear to indicate, he suggested, that the tectum is the “primary driver of the mesencephalic reticulum” (which fits with the evidence for a collicular role in cerebral activation cited above, Jefferson, 1958; Dean et al., 1991; Dringenberg et al., 2003).

There were, however, considerable differences among Denny-Brown's five animals in the nature and severity of their symptoms, extending to the details of their visuomotor behavior. These differences presumably are related to differences in the extent and location of the lesions. The lesions were large and deep, variously encroaching on neighboring structures. In this connection it is worth noting that the behavioral effects of complete and selective lesions of the periaqueductal gray matter are more drastic versions of the kind of global behavioral changes reported by Denny-Brown (see Bailey and Davis, 1942, 1944). It seems plausible, therefore, that these symptoms, including persistent mutism (Gruber-Dujardin, 2010)^5^, relate to damage extending beyond the colliculus into the immediately underlying periaqueductal gray matter or its efferent fibers. In addition, periaqueductal loss of its collicular input (Mantyh, 1982, 1983) may have contributed to the observed deficits.

Perhaps in cognizance of the likelihood that the behavioral symptoms he described involved damage to more than the superior colliculus *sensu stricto*, Denny-Brown ended his lecture on a cryptic note. The periaqueductal gray and above all its “more differentiated peripheral layers,” namely midbrain reticular formation and tectum are vital, he wrote, for unitary functioning of the organism in relation to its surroundings, and constitute what he called *the physiological “ego*.*”* He did not elaborate on this obscure formulation, but this is the first time a linkage between the neural machinery in the roof of the midbrain and “the self” appears in print. Fifteen years later a similar suggestion, focused on the sense of continuity of self over time, is made by the Scheibels with regard to the deeper layers of the superior colliculus and nucleus cuneiformis beneath its caudal border (Scheibel and Scheibel, 1977). They, as well as Denny-Brown, are cited in their turn at late points in an expansive discourse on a collicular locus of “awareness of self” published by the biochemist and gerontologist Bernard Strehler 14 years after the Scheibels (Strehler, 1991).

Of these three, only Strehler attempts a detailed justification of a collicular role in the domain of self and awareness. However, the terminology he applies to this end is so varied and imprecise as to leave the attempt under-constrained from the side of the proposed function. The latter might, by close reading, be narrowed down to “awareness of self-vs-environment” or a system's “cognizance of its own existence” (Strehler, 1991, p. 81). In present terms, these expressions refer to particular contents of consciousness (i.e., cognizance of the distinction between self and environment, or of the fact that one exists, both of which are cognitive contents). They do not, in other words, define factors constitutive of the state that allows contents to be consciously apprehended; rather they presuppose it. If instead we ask whether there might not be some construal of the term self that might in fact refer to a constitutive factor of the conscious state, and how such a factor might be neurally implemented, a possible role for the superior colliculus in the constitution of the conscious state does indeed come within view.

## The self that is excluded from but presupposed by the contents of consciousness

The entire content of our sensory experience bears witness in multiple ways to the egocentric geometry of its spatial arrangement. As far as immediate sensory experience goes, all its contents, irrespective of modality, are arrayed around an approximation to a single point, the point “from which” they all are experienced, be they near or far, high or low, left or right, in front or behind (e.g., sounds). In fact, these very terms are defined in relation to that point, and have no meaning apart from it; the same applies to “sidedness” and “handedness” (James, 1890, p. 150, footnote 2). It is this egocentricity of sensory experience—the fact that visual (as other) objects are perceived from a point—that occasions the occlusion of one visual object by another. In the sense of touch the sensation of a light touch to a finger is experienced as located in the finger, but that sensation *in* the finger is not experienced *from* the finger, but from about the same spatial location from which that finger is seen, even if the sensation should occur in pitch darkness. Our spatial senses are integrated, in other words, into a single, panoramic multimodal space anchored to its egocenter common origin (see Figure 1)^6^.

That point, that origin, lies at the intersection of all lines of sight, serving as their common pivot (cf. Vetter et al., 1999; Wagner, 2006; Thaler and Todd, 2009). It is located at the proximal-most end of any line of sight or equivalent line of attentional focus (say for somesthesis in the dark). It is the “here” with respect to which any sensory (or other) percept is “there.” It is the point, in other words, from which we are looking and, more generally, registering sensory experience or deploying attention. For our visual perception of the world, that point can be determined with millimeter precision by a simple procedure first developed by Hering (1879/1942; Roelofs, 1959). Commonly included in lab exercises in the psychology of perception it empirically pinpoints the intersection of a few lines of sight obtained by fixating specified environmental locations and aligning fiduciary pins with them along each of the lines of sight (Howard and Templeton, 1966).

**Figure 4:**
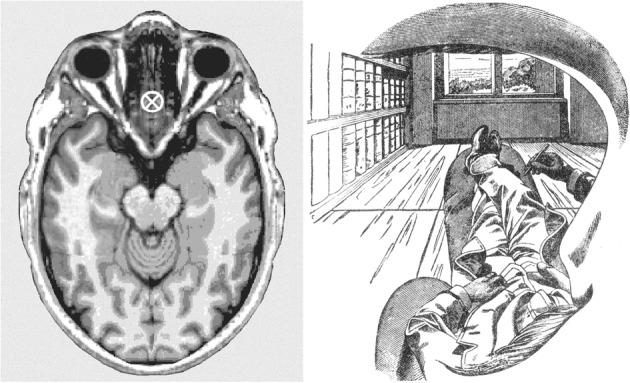
**Left panel:** The present author's visual egocenter, empirically determined by the method of Hering (Howard and Templeton, 1966), and transferred onto a horizontal structural magnetic resonance image of a human head at the level of the eyes, where it is marked by a cross (Image, ^©^Nevit Dilmen found at Wikimedia commons, released under a Creative Commons Attribution-Share Alike 3.0 Unported license). **Right panel:** A monocular view from the visual egocenter, rendered by Ernst Mach through his left eye (Mach, 1897, Figure 1, p. 16). The dark fringe of Mach's eyebrow appears beneath the shading in the upper part of the figure, the edge of his moustache at the bottom, and the silhouette of his nose at the right-hand edge of the drawing. These close-range details framing his view are available to our visual experience, particularly with one eye closed, though not as crisply defined as in this drawing. In a full cyclopean view with both eyes open the scene is framed by an ovoid within which these proximal details typically disappear from view. Apparently Mach was a smoker, as indicated by the cigarette extending forward beneath his nose. Digitally retouched version of Mach's drawing reproduced courtesy of Wikimedia (http://commons.wikimedia.org/wiki/File:Ernst_Mach_Innenperspektive.png. Note the apparent impossibility of having an unobstructed view of a scene from the empirically determined point marked on the image on the left, a point which is surrounded on all sides by biological tissues (see further the text).

Thus, determined, the visual egocenter is found to be, first of all, single (not a foregone conclusion given that we have two eyes) and it turns out *not* to be located, as one might suppose, at the midpoint between the centers of rotation of the two eyes. Rather, it lies deeper inside the head, in the midsagittal plane, some 4–5 cm behind the bridge of the nose (see left panel, Figure 4). This empirically determined location inside the head from which we look out upon the world along straight and uninterrupted lines of sight is of course surrounded on all sides by biological tissues. Here lies the ultimate conundrum of phenomenal sensory awareness, the Achilles heel of its secret, in fact. How it is possible to have unobstructed lines of sight into the world from a place inside our heads that is surrounded on all sides by opaque tissues?

The short answer is that our experienced head is the head of the neural reality model (see Figure 1, rastered head), for which arrangements are possible that are not realizable in the physical head itself. For details, see my previous publications (Merker, 2012, pp. 53, 55 and 2013, pp. 26–27). Here, we are concerned, rather, with what it is that occupies this enigmatic location at the origin of the line of sight.

Typically our line of sight is deployed to a distal object of interest, but let us reverse the direction of our interest by “moving backwards” along a line of sight toward its proximal origin. We will then traverse a succession of environmental locations ever closer to ourselves, to arrive in the vicinity of our eyes. At these close quarters we may espy the shadowy presence of the edge of our orbit in peripheral vision, particularly if, as in Figure 4, we follow Ernst Mach's example, and close one eye. Then, as we try to proceed all the way to the origin of the line we have followed, an origin we know to be located inside our head, we are suddenly at a loss for any determinate content of consciousness whatsoever that might inform us about the nature of that which occupies the origin of the line we have followed backwards. Disappointed, but not defeated, we press on, and continue progress along the extension of the line of sight through the troublesome lacuna we landed in, to have our focus arrive in short order at the back of our head.

We are then free to continue our imaginary journey out into the world behind our head. There is, however, no need to do so, because the answer to the question of what occupies the origin of the line of sight is already at hand. For every step away from the troublesome lacuna, even to a distance as short as to the back of our head, the points along the line we are tracing are ever more distant from the place from which we are conducting the exercise. We are, in other words, increasing the distance between our targets and ourselves, in a reverse motion from the one that brought us to the lacuna. What occupies the lacuna, then, can be nothing other than we ourselves. The place from which one is looking or attending is occupied—necessarily, unsurprisingly, and tautologically—by oneself.

This “oneself,” the self thus located through the above first person exercise, is not and cannot be a self-image of any kind. It defines the viewpoint from which any and all images are viewed—or equivalently, is the origin of all lines of sight (and “lines of attention,” the exercise was conducted by covert attention). It is the one location that is forever beyond the reach of any directed attention or perception, because it is the point from which attention is directed and relative to which percepts are located in the space whose origin it defines.

This helps explain the utter blank one draws in attempting to take the last step along the line of sight back to its origin. That location is excluded from the contents of consciousness by the same geometric necessity that prevents an eye from viewing itself, though it is the instrument for viewing all else (Schopenhauer,1844/1958, vol. 2, p. 491; see also Baars, 1988, pp. 327ff for “contextual” aspects of consciousness). This is what David Hume failed to realize when he “searched his mind” for a self and found only perceptions and bundles of perceptions (Hume, 1739/1888). The self he was looking for is the place from which he was looking.

The first person exercise we have just conducted yields a minimal definition of the self as the perceptual egocenter of sensory consciousness and, by extension, of all awareness. It defines a location with respect to which any and all conscious percepts can be uniquely localized in space by direction and distance relative to that point. Some of these percepts are located inside our skin—say, a stirring of joy in our breast or a headache—yet they are still perceived relative to that self-same egocenter. Its location inside the head just behind the eyes—a convenience for the control of orienting movements, as we have seen—is in good agreement with our intuitive sense of “where we (and others) are located” as recently determined empirically by a third-person procedure. Both children and adults assign that location to the vicinity of the eyes (Starmans and Bloom, 2012).

For present purposes, it matters little whether that assignment draws on first person intuitive conclusions along the lines of our exercise above, or on the sense that the lively play of a person's eyes bear more immediate and direct witness to their interests and intentions—and hence to their self—than do other visible behaviors. Perhaps it is a combination of both, because the two are intimately related. When, for purposes of the above exercise, we moved attention along our line of sight we were doing no more than making deliberate use of the routine functional role of our perceptual vantage point (egocenter) in directional movements of attention and gaze. It is only in relation to the perceptual egocenter that the size and direction of the angular displacement required of a given gaze or attentional movement are defined. As the implicit reference of all such movements it is the central functional pivot from which they issue, not as motor instructions for a particular combination of eye, head and trunk movements, but rather as locational pointers to targets in egocentric space to be attained by the very next orienting or attention movement.

But that is reminiscent of the function attributed to the superior colliculus in the previous section. Might this midbrain structure in fact—as first suggested in the vaguest of terms by Denny-Brown—serve as the physiological “ego” or self in the minimal sense just outlined? The exercise which led us to this possibility provides an initial plausibility check on whether it might do so. That exercise was conducted by directing attention alone, without eye or head movements, forwards and backwards from the egocenter lacuna, i.e., by *covert* attention in a full 360 degree egocentric space. The involvement of the superior colliculus in covert spatial attention is well established (Robinson and Kertzman, 1995; Cavanaugh and Wurtz, 2004; Ignashchenkova et al., 2004; Muller et al., 2005; Fecteau and Munoz, 2006; Lovejoy and Krauzlis, 2010; Schneider, 2011). Does it also host a full 360 degree directional compass, without which it could not have allowed us to move covert attention to the back of our head, and without which it cannot serve as central pivot or origin of a fully functional multimodal and egocentrically organized localization system (see Figure 1, and below)?

When animals are free to move their head, collicular stimulation at increasingly caudal levels evokes increasingly extensive gaze excursions beyond the oculomotor range by recruiting ever larger head movements into the orienting response (Faulkner and Hyde, 1958; Westheimer and Blair, 1975; Roucoux et al., 1980; King et al., 1991; Grantyn et al., 1992; Freedman et al., 1996; Sparks, 1999; Corneil et al., 2002; Isa and Sasaki, 2002; see also Guitton and Volle, 1987). For natural orienting movements into the space behind the animal, head turns by means of cumulative rotation across increasingly caudal cervical vertebrae (Richmond et al., 1992) are supplemented by trunk movements (Hassler and Hess, 1954). The same recruitment of eyes, head and trunk by collicular stimulation is true of non-mammals (Herrero et al., 1998; Saitoh et al., 2007). Since, as already noted, the details of movement execution are left to brainstem structures downstream of the colliculus, the colliculus itself appears to implement a space of pure locational specification for the entire egocentric surround^7^.

With a full collicular complement of spatial directionality, the path is cleared for the possibility that this midbrain structure in fact occupies the position in the neural machinery of the brain that gives us our position as first person inhabitants of an egocentrically organized space of phenomenal sensory awareness, while it itself lies outside the compass of phenomenal awareness. As already detailed, though that position is the defining feature of such a space, it cannot itself appear as a phenomenal content within it. In fact, all phenomenal contents, as we have seen, are separate from it, because that location defines the ultimate unobservable “here” with respect to which they are located “there.” If the superior colliculus in fact implements the directional pivot—an omnidirectional non-phenomenal “here” for all phenomenal “theres”—how and where are those phenomenal contents implemented, and how is the colliculus related to that larger arrangement of which it must, on this interpretation, form an integral part?

## Tethering phenomenal space to its non-phenomenal directional pivot

In view of all that has gone before, only two possibilities remain: the space within which sensory information achieves conscious status, i.e., phenomenal space, is implemented either within the colliculus itself or among the targets of its ascending projections. Regarding the first alternative, the multimodal laminar colliculus features every modality on which animals rely for their phasic sensory orienting. This includes exotic ones in some species, such as infrared (Hartline et al., 1978), electroceptive (Bastian, 1982), magnetic (Nemec et al., 2001), and echolocation senses (Valentine and Moss, 1997). These modal maps, layered cortex-like through the collicular/tectal depth dimension (see Figure 3), share the collicular efferent premotor functional framework in its tangential dimension. In the collicular output layers its multiple modalities converge onto single collicular neurons with cortically dependent multimodal properties (Meredith et al., 1992; Wallace and Stein, 1994). Moreover, collicular neuron numbers would seem to suffice for implementation of a comprehensive multisensory phenomenal space. A total (bilateral) neuron count of almost 2 million for the macaque superior colliculus (Théoret et al., 2001) can be extrapolated to about 5 million for the human. This, according to the rough estimate provided in an earlier section, should suffice for the purpose.

There are good reasons, nevertheless, to discount the colliculus as a serious contender for the honor of hosting our phenomenal sensory consciousness. The phenomenal world we inhabit is not only crowded with intricate pattern detail, but brightly colored and exquisitely articulated in its depth dimension both in terms of global spatial relations and solid object shapes. The neural operations of the superior colliculus, on the other hand, seem concerned primarily with locational matters, to the exclusion of much of this intricate and gaudy finery (but see Rizzolatti et al., 1980). Thus macaque collicular single units dispense with the orientation and directional specificity carried by axons of its visual cortical afference, presumably by convergence of multiple differently tuned cortical afferents onto single collicular units, rendering them broadly tuned or untuned (Finlay et al., 1976).

Regarding the color *selectivity* which is an absolute requirement for implementing human phenomenal contents, the colliculus appears to lack it. Its direct retinal afference proceeds from broad-band retinal ganglion cells, and the indirect pathway to the colliculus via the lateral geniculate and primary visual cortex appears likewise to be a broadband, magnocellular pathway lacking color selectivity (Schiller et al., 1979). That does not mean that collicular units lack color *sensitivity*, however: they respond vigorously to stimuli defined by isoluminant color patches alone, but they do so without discriminating stimulus wavelength (White et al., 2009). This color-based information appears to arrive at the colliculus from extrastriate sources, again presumably by convergence of color tuned units. This allows the colliculus to respond to colored stimuli without representing their hue. Such an arrangement fits well with its localizing function but badly with a venue hosting multi-colored phenomenal space.

Regarding three-dimensional depth, finally, the situation is less clear. It hinges on the thorny issue of whether or not collicular output is a purely directional (“cyclopean”) signal, or includes a vergence and torsional signal for the alignment of the two eyes (van Opstal et al., 1991; Chaturvedi and Van Gisbergen, 1999; Walton and Mays, 2003; Busettini and Mays, 2005; Waitzman et al., 2008; Pérez Zapata et al., 2013). The parietal gaze area transmits disparity information to the superior colliculus (Gnadt and Beyer, 1998), yet collicular disparity sensitive units are broadly tuned (Berman et al., 1975; Dias et al., 1991; Bacon et al., 1998), perhaps again reflecting collicular pooling of cortical specificities. These units have been found in the rostral colliculus, where fixation units are also found, so possibly they play a role in fixation behavior. In view of the negative evidence reported for torsion by van Opstal and colleagues and for vergence by Walton and Mays (cited above), there would not seem to be a strong general case for a collicular “third dimension.”

Taken together, these several strands of evidence regarding collicular single unit properties weigh against a collicular locus for full, ordinary phenomenal sensory experience. The process of elimination therefore leaves only targets of ascending collicular projections to consider as possible candidate sites for colliculo-phenomenal interaction. Recall that the search is for a subcortical target of cortical layer V projections capable of relieving the cortex of the need to precipitate a global best estimate of sensory circumstances within cortical probabilistic operations themselves. That target has now been further specified “from below” as a target of ascending collicular projections, and these are concentrated to the thalamus (see Figure 2 and the text it illustrates).

Two further requirements must be fulfilled for a structure to serve the cortex as its global best estimate buffer. It must be reciprocally connected with a broad range of cortical areas occupying the higher levels of the several cortical sensory hierarchies, and must contain the intrinsic circuitry needed to conduct swift multiple constraint satisfaction operations over these cortical afferents in the span of the few hundred milliseconds available between gaze shifts (Rayner, 1998; see Merker, 2012, p. 56 for details). The constraint satisfaction operation accordingly must be conducted in parallel fashion (cf. Mezard and Mora, 2009) through interactions beyond strictly local ones in the candidate structure. Generally, however, the thalamus is conspicuously lacking in intrisic connectivity within or between its subdivisions (e.g., Trojanowski and Jacobson, 1975; Ogren and Hendrickson, 1977). It therefore lacks a crucial anatomical requirement for implementing the needed constraint satisfaction operation. There is, however, one notable exception to this generalization.

The dorsal pulvinar of the higher-order thalamus is a multimodal region connected with high level posterior parietal and temporal areas of both streams of the visual system, with auditory association cortex and multimodal cortical areas, as well as with parahippocampal, prefrontal (including frontal eye fields), orbitofrontal, and insular cortices (Yeterian and Pandya, 1991; Gutierrez et al., 2000; Imura and Rockland, 2006; Kaas and Lyon, 2007; see also Cappe et al., 2009). The caudal reaches of this dorsal pulvinar territory are invested with a unique population of long range inhibitory interneurons (Imura and Rockland, 2006). Their axons branch widely across the many intricately interdigitated slabs or discs by which cortical areas are represented there (e.g., Asanuma et al., 1985; Hardy and Lynch, 1992). Though connective detail is as yet lacking, these axons, being inhibitory, can hardly avoid establishing competitive linkages and bridges across these interdigitated slabs. The reach of these inhibitory interneurons within the dorsal pulvinar is extra-local but *less than global* (see insets in Figures 5, 6, and 8 of Imura and Rockland, 2006). Unlikely, therefore, to operate as a winner-take-all decision mechanism, this inhibitory cross-connectivity may instead constitute a powerful means of swift multiple constraint satisfaction over the interdigitated mosaic of the cortical areas represented there (see also Imura and Rockland, 2007).

This is also the part of the pulvinar that features neurons that combine selectivities of both the dorsal and ventral streams of the visual system in single neurons (Benevento and Port, 1995), that show *more selectivity for stimulus awareness than cortical visual areas assessed with the same method* (Wilke et al., 2009; see also Padmala et al., 2010), that correlate with confidence in sensory judgments (Komura et al., 2013), that reflect intentional rather than routine movements (Acuña et al., 1983), and whose reversible inactivation disrupts selection of action plans (Wilke et al., 2010). The powerful influence of pulvinar activity over the visual responsiveness of even V1 neurons is also worth noting (Purushothaman et al., 2012), as is the longstanding association of the pulvinar with sensory attention and neglect (Petersen et al., 1987; Karnath et al., 2002; Rushmore et al., 2006; Saalmann et al., 2012). Though it does not, of course, prove it, all of this fits well with the conjecture that the dorsal pulvinar implements the brain's global best estimate of sensory circumstances in temporary buffer fashion (further circumstantial evidence bearing on this identification is available in Merker, 2012, pp. 63–69).

Proceeding, then, on the working hypothesis that the dorsal pulvinar in fact performs this best estimate buffer function, it remains to consider how the “first person” might enter its operations. In the preceding section, this inherent aspect of sensory consciousness was found to be implicated in the directional function of covert and overt orienting by defining its implicit (non-phenomenal) spatial origin. This suggested the collicular priority gate, with its omni-directional orienting system, as a candidate implementing structure. It can be related to the dorsal pulvinar via the connectivity depicted in Figure 2, by noting that the principal elements and connections of Stewart Shipp's proposed functional anatomy of the brain's attention system lie embedded in that connectivity (see Shipp, 2003, 2004). In his scheme, the *ventral* pulvinar fills the role of principal “salience map” (Shipp, 2004, Figures 2a,g). However, to fill that role it would need intrinsic circuitry by which to crown a “winner” among alternate bids for target priority among its stacked visual topographies, yet in keeping with thalamic patterns generally, this pulvinar subdivision presumably lacks such circuitry.

The functional logic of Shipp's scheme survives this problem, however, because the requisite circuitry is available in the superior colliculus, as we have seen. The colliculus is an integral part of his scheme, and can therefore substitute in it for the ventral pulvinar as principal “salience map” (“priority gate” in present terms). A collicular rather than ventral pulvinar locus also has the advantage that it generalizes priority selection across all spatial modalities (instead of being confined to vision alone), as it must in order to qualify as a general spatial attention system. Moreover, as “orienting super-hub” the colliculus engages principally when alternative bids from a variety of sources, not least cortical, compete for the location of the target of an orienting or attention movement. On the present account such competition is settled within the collicular circuitry itself, and in its deeper layers in final terms. They are therefore the first site in the brain to “know” which location will be the target of the next saccadic gaze shift *actually to be executed*, and thus ideally situated to convey this decision to the forebrain via their ascending projections to the thalamus.

What is conveyed to the forebrain in this way, then, can be nothing other than the predictive “attention pointers” proposed to prepare forebrain sensory maps for impending gaze shifts peri-saccadically (for which see Wurtz, 2008; Cavanagh et al., 2010; also Hulme et al., 2010; Prime et al., 2011). Given that even *top-down* biasing of covert attentional selection in a distractor task requires an intact superior colliculus (Lovejoy and Krauzlis, 2010), the predictive pointer function presumably is the phasic variant of a more general overt and covert directional orienting signal conveyed to the forebrain from the colliculus via its ascending projections. From there it propagates as a local attentional bias shared by all relevant forebrain maps on account of the topographic matching of their connectivities across telencephalic, diencephalic, and mesencephalic levels, exactly as detailed in the Shipp model of the attention system (Shipp, 2004).

The answer is now at hand to the question of how a “collicular self,” construed as a non-phenomenal directional pivot for phenomenal sensory space, might relate to the proposed implementation of that space in the dorsal pulvinar. First, the dorsal pulvinar receives direct projections from the superior colliculus, originating—as they should, according to the above—in the deeper collicular lamina (Benevento and Standage, 1983). Second, all the gaze-related areas in cortex and basal ganglia that receive the collicular signal via the extended intralaminar complex and higher-order thalamus are bound to reflect the play of the collicular attention/orienting pointers in their operations.

The incessant play of these pointers will therefore figure as one of the variables in the massive operation of probabilistic source reconstruction in which the cortex is permanently engaged, both to decipher the immediate sensory situation it faces from moment to moment, and for the cumulative (learned) acquisition of the prior competence with which it meets that challenge. This prior competence will therefore inevitably come to reflect the invariant behind the play of the directional attention/orienting pointers, namely the point of origin with respect to which their directional differences are defined. If the primary function of the dorsal pulvinar is indeed mutual constraint satisfaction across its diverse afferents, then the resulting global best estimate of sensory circumstances it produces will come to incorporate this invariant embedded in its cortical afference, complemented by collicular afference from below. It will figure there as exactly what in fact it is, a tacit perspective point *implicit* in the perspectival organization of the phenomenal contents of the global best estimate sensory buffer, without being present as a phenomenal object in it.

This point, then, which is the point from which we look and feel, is our tacit first person perceptual egocenter or self. It is only the innermost of the similarly extracted invariants behind the clusters of correlated variances which our receptor surfaces present to the brain for disambiguation, and which in their momentary global best estimate form we experience as our body and the world which surrounds it (Merker, 2012, p. 54; see also Philipona et al., 2003, 2004). As a product or derivative of the lively play of collicularly triggered orienting and attention movements, the orienting superhub in the roof of the midbrain is its ultimate anatomical base. The decision making machinery hypothetically incorporated into the schematic egocenter in my previous publications (see Merker, 2012, pp. 59, 68; Merker, 2013, pp. 19–22, and Figures 1.2 and 1.4 in particular) accordingly is the intrinsic collicular circuitry by which the priority target of the very next orienting or attention movement is settled.

In the scheme proposed here, this ultimate collicular pivot of the mechanism of consciousness lies outside the anatomical structure implementing conscious contents. This provides a felicitous fit with the phenomenal inaccessibility not only of the self that anchors the first person perspective in which alone those contents come to us in consciousness, but also with our lack of conscious access to the continual split-second decision-making by which it expresses itself in the incessant movements of our gaze across its targets.

## Conclusion

To summarize, the movements of our gaze or attention from a point inside the nested structure of body within world that is our phenomenal sensory space supplies the leading edge of practically all our behavior. Moving from target to target, it precedes our instrumental engagement with the world like the acquisition marker of a laser spotter in a combat zone. The point from which the pointer proceeds is thus not only the tacit perceptual egocenter or self, it is also, and without the need to make additional assumptions, the central pivot of action control. This, then, is the burden of the present bid to naturalize the first person perspective in action control by assigning a role, in the functional economy of the brain's efference cascade, to our tacit sense of occupying a place inside our heads from which we survey our world and direct the movements of our body within it.

### Conflict of interest statement

The author declares that the research was conducted in the absence of any commercial or financial relationships that could be construed as a potential conflict of interest.

## Footnotes

^1^Unless, of course, a cortical area needs to utilize the highly specialized motoric capacity for which primary motor cortex appears to have evolved, the control of that fraction of behavior that consists of the skilled (learned) and detailed patterning of movements of distal extremities, or effectors such as those involved in vocal learning (Heffner and Masterton, 1975; Lawrence and Hopkins, 1976; Passingham et al., 1978; Kuypers, 1981; Karni et al., 1998; Rathelot and Strick, 2006; Okanoya and Merker, 2007; Brown et al., 2008; Lemon, 2008).

^2^One possible reason for this neglect is described in the final section of Merker (2013). In brief, it may betoken a lingering and entirely tacit influence of naive realism on theorizing such that this world that surrounds us is not recognized as a content of consciousness but is mistaken for the actual physical universe itself. Such misattribution eliminates a major portion of the contents of sensory consciousness from consideration vis-a-vis consciousness theory, whose purview accordingly shrinks to matters of our “inner life,” thinking, self-consciousness, qualia, and the like.

^3^Recognition of this collicular function was long delayed by the fact that the head of the experimental animal was fixed in a number of key physiological experiments designed to probe collicular function, and a restricted set of stimulation sites and parameters in early experiments in which the animals were free to move their heads (Robinson and Jarvis, 1974; Stryker and Schiller, 1975; see further Sparks, 2004). With less restrictive experimental conditions, not only does collicular stimulation evoke integrated gaze movements combining movement of eyes and head (Freedman et al., 1996; Sparks, 2004), but the animals' localization ability is drastically improved (Tollin et al., 2005), and the relationship between collicular unit activity (as well as stimulation site) and behavior is altered (see Sparks, 1999 for details).

^4^In the author's studies of escape behavior in hamsters (Merker, 1980), frame-by-frame analysis of the filmed trials showed that no orienting movements toward the over-head sudden silent visual threat preceded the explosive escape behavior triggered by the stimulus (Merker, 1980). Instead the animal instantly reoriented to one of two escape routes in the familiar testing arena, and scrambled to safety, a behavior that was abolished by undercutting the superior colliculus, severing its descending projections. In present terms, on the rare and sudden appearance of a large, dark, and silently but swiftly moving visual stimulus in the animal's upper visual field the location of an escape route, known to the animal from long established familiarity with the testing arena, became its compelling “spatial target of immediate behavioral priority.” For effective visual threats in rodents, see Wallace et al. (2013).

^5^It appears that the integrative role of the periaqueductal gray in vocal behavior [for which see review by Gruber-Dujardin (2010)] in fact resembles the role here proposed for the colliculus in orienting behavior.

^6^Visual experience is panoramic: no one has ever experienced that mainstay of philosophical discussions of perception—the “red of a tomato”—in itself and as such, but always only in a particular location with a visual surround, typically rich in other objects arrayed around it and all of them together around us. It is an egregious error to imagine that the problem of perception can be approached by “starting simple” to build complexity from elementary sensations (the tortuous nature of William James' attempt to do so is a case in point; James, 1890, pp. 145–166).

^7^The primitive position of the eyes in vertebrates is lateral, on the sides of the head, a placement exhibited by most non-mammalian and many mammalian species. Such animals have visual fields that essentially cover their full surround. The colliculus has no “reason” to contract its full field sensorimotor organization in the minority of species whose eyes have migrated to a frontal position. With frontally placed eyes, head movements are required to move the visual field beyond the oculomotor range. By leaving collicular full-field organization intact, head movements can be collicularly triggered now as before by, say, somatosensory or nociceptive stimuli to body parts beyond the reach of vision, or sound sources localized in the rear sector of space (see also footnote 3 and references therein).
